# Chemogenetic Inhibition of the Amygdala Modulates Emotional Behavior Expression in Infant Rhesus Monkeys

**DOI:** 10.1523/ENEURO.0360-19.2019

**Published:** 2019-10-11

**Authors:** Jessica Raper, Lauren Murphy, Rebecca Richardson, Zoe Romm, Zsofia Kovacs-Balint, Christa Payne, Adriana Galvan

**Affiliations:** 1 Yerkes National Primate Research Center, Emory University, Atlanta, Georgia 30329; 2 Department of Pediatrics, School of Medicine, Emory University, Atlanta, Georgia 30329; 3 Department of Psychology, Emory University, Atlanta, Georgia 30329; 4 Drexel University, Philadelphia, Pennsylvania 19104; 5 Marcus Autism Center, Atlanta, Georgia 30329; 6 Department of Neurology, School of Medicine, Emory University, Atlanta, Georgia 30329

**Keywords:** attention, development, DREADDs, emotion, nonhuman primate, social

## Abstract

Manipulation of neuronal activity during the early postnatal period in monkeys has been largely limited to permanent lesion studies, which can be impacted by developmental plasticity leading to reorganization and compensation from other brain structures that can interfere with the interpretations of results. Chemogenetic tools, such as DREADDs (designer receptors exclusively activated by designer drugs), can transiently and reversibly activate or inactivate brain structures, avoiding the pitfalls of permanent lesions to better address important developmental neuroscience questions. We demonstrate that inhibitory DREADDs in the amygdala can be used to manipulate socioemotional behavior in infant monkeys. Two infant rhesus monkeys (1 male, 1 female) received AAV5-hSyn-HA-hM4Di-IRES-mCitrine injections bilaterally in the amygdala at 9 months of age. DREADD activation after systemic administration of either clozapine-*N*-oxide or low-dose clozapine resulted in decreased freezing and anxiety on the human intruder paradigm and changed the looking patterns on a socioemotional attention eye-tracking task, compared with vehicle administration. The DREADD-induced behaviors were reminiscent of, but not identical to, those seen after permanent amygdala lesions in infant monkeys, such that neonatal lesions produce a more extensive array of behavioral changes in response to the human intruder task that were not seen with DREADD-evoked inhibition of this region. Our results may help support the notion that the more extensive behavior changes seen after early lesions are manifested from brain reorganization that occur after permanent damage. The current study provides a proof of principle that DREADDs can be used in young infant monkeys to transiently and reversibly manipulate behavior.

## Significance Statement

Many neurodevelopmental disorders exhibit abnormal structural or functional amygdala development and altered socioemotional behavior. To date, developmental neuroscience studies have relied on permanent lesion techniques to investigate how atypical amygdala development impacts socioemotional behaviors, which may not adequately recapitulate the role of amygdala dysfunction in the manifestation of aberrant behavior. The present study sought to demonstrate that chemogenetic techniques based on designer receptors exclusively activated by designer drugs (DREADDs) could be used to transiently inhibit amygdala activity in infant monkeys, resulting in alterations in socioemotional behavior. This proof-of-principle study supports the use of chemogenetics for developmental neuroscience research, providing an opportunity to broaden our understanding of how changes in neuronal activity across early postnatal development influences behavior and clinical symptoms.

## Introduction

The amygdala plays an important role in social behavior and emotional responses to threats in the environment (Aggleton, 2000). This knowledge has largely come from research on adult humans and animals. Yet, the amygdala has a protracted postnatal development (Payne et al., 2010; Chareyron et al., 2012; Hunsaker et al., 2014), in which growth of the amygdala appears to coincide with the refinement of social and threat-detecting skills (Mendelson, 1982; Mendelson et al., 1982; Kalin et al., 1991). Investigations of how amygdala development contributes to these skills have largely used permanent lesion techniques, finding that damage to the amygdala during the early postnatal period contributes to life-long changes in social, emotional, and neuroendocrine function (Bauman et al., 2004; Bliss-Moreau et al., 2010, 2011a,b, 2017; Raper et al., 2013b, 2014a,b; Stephens et al., 2015; Moadab et al., 2017). Given the potential for neural plasticity during this postnatal period, there is considerable opportunity for reorganization and compensation from other brain structures. In fact, several studies have demonstrated how lesions early in life contribute to such reorganization (Machado et al., 2008; Raper et al., 2014b; Grayson et al., 2017; Payne et al., 2017). To avoid the pitfalls of permanent lesions and better address these questions, we need tools that transiently activate or inactivate the amygdala during early development.

Pharmacological inactivation techniques have been used to manipulate amygdala activity and influence behavior (Wellman et al., 2005, 2016). However, these techniques require the surgical placement of skull-mounted chambers for intracerebral drug infusions, which are not ideal for developmental research on infant monkeys with rapidly growing brains and bodies. An alternative approach that would not require permanently mounted hardware or rely on repeated intracerebral injections is greatly needed for developmental research.

Chemogenetic techniques provide powerful tools for behavioral neuroscience because they allow remote manipulation of neuronal activity (Sternson and Roth, 2014). Designer receptors exclusively activated by designer drugs (DREADDs) are the most commonly used chemogenetic tool. DREADDs are synthetic variants of G-protein-coupled muscarinic receptors (Armbruster et al., 2007; Farrell and Roth, 2013; Roth, 2016), which are not activated by acetylcholine (the endogenous ligand of muscarinic receptors) and are not constitutively active. Instead, DREADDs are exclusively activated with high efficacy by exogenous ligands. The available DREADDs include receptors that are coupled to G_i/o_, G_q/11_, and G_s_ proteins termed M4Di, M3Dq, and GsD, respectively (Wess et al., 2013; Roth, 2016), providing tools that can either increase or decrease the activity of transduced neurons, depending on the intracellular pathway activated. The expression of DREADDs can be targeted to specific neurons using viral transfection (Urban and Roth, 2015; Roth, 2016). The most commonly used ligand to activate DREADDs is clozapine-*N*-oxide (CNO), although recent reports have shown that CNO can be reverse metabolized into clozapine (Raper et al., 2017; Allen et al., 2019). Thus, an alternative method to activate the DREADDs is to use clozapine itself, at low doses that will only preferentially activate the DREADDs (Gomez et al., 2017).

DREADDs have been widely used in rodent models, but reports in nonhuman primates (NHPs) have been limited. To date, only four publications have reported DREADDs manipulation of behavior or neuronal activity in NHPs (Eldridge et al., 2016; Grayson et al., 2016; Nagai et al., 2016; Upright et al., 2018). While these studies provide critical information demonstrating the ability to use chemogenetic tools in NHPs to manipulate neuronal activity and impact behavior, they have all focused on adult monkeys. Considering its advantages over permanent lesions and other transient inactivation techniques (i.e., reversibility and minimal invasiveness), chemogenetics hold their greatest promise for developmental neuroscience research. The current study provides proof of principle that chemogenetic tools can be used to manipulate amygdala activity in infant monkeys.

## Materials and Methods

### Subjects

**Figure 1.**
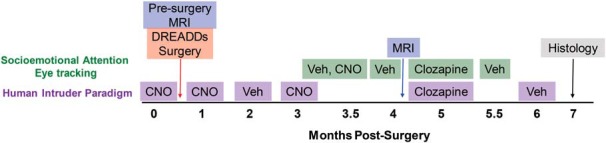
Schematic of the study design. Two infant rhesus monkeys received bilateral injections of AAV-hM4Di DREADD into the amygdala at 9 months of age, followed by 6 months of testing with either Vehicle (Veh), CNO (10 mg/kg), or clozapine (0.1 mg/kg). Before DREADD transduction, infants were tested on the human intruder paradigm with CNO to rule out any impact of the drug on the behavior in naive animals. MRI was conducted immediately before surgery to aid with amygdala injection localization and again at 4 months postsurgery in relation to another study not reported here. Monkeys were killed at 7 months postsurgery to determine DREADD expression with histology.

Two infant Indian rhesus macaques (*Macaca mulatta*), one male and one female (1.85 and 2.0 kg body weight, respectively), were used in this study. The animals were raised by their mothers in large social groups at the Yerkes National Primate Research Center (YNPRC) Field Station (Lawrenceville, GA) until 7 months of age, when they were transferred to indoor pair housing at the YNPRC Main Station (Atlanta, GA) for the duration of the study. The Animal Care and Use Committee of Emory University approved all procedures, which were performed in accordance with the National Institutes of Health *Guide for the Care and Use of Laboratory Animals*. Figure 1 outlines the study design, behavioral testing, and timing of ligand administrations.

### Screening for adeno-associated virus neutralizing antibodies

The native immunity of nonhuman primates has been shown to impact transduction in other CNS tissues (i.e., eye; Kotterman et al., 2015) and neutralizing antibody response to multiple virus solution intracerebral injections have been directly linked to weak transduction in neurons (Mendoza et al., 2017). However, it is currently unclear whether native immunity to adeno-associated viruses (AAVs) can impact the transduction of neuronal tissue. Out of an abundance of caution, infant monkeys were screened for neutralizing antibodies to AAV5 before surgery to reduce the possibility that such antibodies might interfere with viral transduction.

Blood samples (2 ml) were collected in tubes containing EDTA (3.5 mg), were centrifuged at 2500 relative centrifugal force (rcf) for 15 min, and plasma was pipetted off for assay by the Virology Core at the YNPRC (Atlanta, GA). AAV5 neutralization titers were determined by IC_50_ on HeLa cells (catalog #CRL-2972, ATCC; RRID:CVCL_B053) freshly infected with adenovirus serotype 5 (Ad5, catalog #VR-5, ATCC). Briefly, plasma samples were heat inactivated for 1 h at 56°C and diluted to 1:5, 1:20, 1:80, and 1:240. Plasma dilutions were then mixed with AAV5 bearing a luciferase expression construct (AAV5-luc, University of North Carolina Joint Vector Laboratory, Chapel Hill, NC; RRID:SCR_002448) and incubated for 1 h at 37°C. After initial incubation, plasma/AAV5-luc mixtures were added to the Ad5-infected HeLa cells and incubated at 37°C in 5% CO_2_ incubator for 48 h. Luciferase expression was determined using the Bright-Glo Luciferase Assay System (Promega) according to the manufacturer protocol and measured on a BioTek Gen5 luminometer. The IC_50_ was calculated relative to negative serum controls. Results revealed that neutralizing antibody titers for AAV5 were below the level of detection in the male and 1:20 for the female.

### Surgery

At 9 months of age, animals underwent DREADD transduction surgery. The injection of inhibitory DREADDs bilaterally into the amygdala followed procedures similar to those described in previous studies of amygdala lesions in monkeys (Raper et al., 2014b). Briefly, on the day of surgery, the infants were removed from their cagemate, sedated with ketamine hydrochloride (1 mg/kg), and maintained with isoflurane (1–2% to effect). Their head was secured in a nonferromagnetic stereotaxic apparatus, and T1-weighted MRI sequences were acquired using a Siemens 3.0 T/90 cm whole-body scanner and a 3 inch circular surface coil. A high-resolution T1-weighted scan [spin-echo sequence: echo time = 11 ms, repetition time = 450 ms, contiguous 1 mm section, 12 cm field of view, 256 × 256 matrix] obtained in the coronal plane was used to determine the coordinates of injection sites in the amygdala. An additional T1-weighted MRI was acquired at 4 months postsurgery in the context of other studies (not reported here), following these same procedures.

All surgical procedures were performed under aseptic conditions, an intravenous drip (0.45% dextrose/0.9% NaCl) was placed to maintain normal hydration, and vital signs (heart rate, respirations, blood pressure, expired CO_2_) were monitored throughout the surgery. Nolvasan solution was used to disinfect the scalp, and a local anesthetic (bupivacaine 0.25% concentration, 1.5 ml) was injected subcutaneously along the midline to reduce the pain during skin incision. After the skin and underlying connective tissue were gently displaced laterally, two small craniotomies were made in front of bregma and above the amygdalae, and the dura was cut and retracted to expose the brain. Animals received viral vector injections of the DREADD construct AAV5-hSyn-HA-hM4Di-IRES-mCitrine by Dr. Bryan Roth [6 × 10^12^ vg (vector genome)/ml; Plasmid #50464, Duke Viral Vector Core, Duke University, Durham, NC; RRID:Addgene_50464] administered in eight sites within the center of each amygdala using a 10 µl Hamilton syringe. Needles were lowered simultaneously in both hemispheres, 5 µl were manually injected (0.4 µl/min) at each site for a total of 40 µl per hemisphere. After each injection, a 3 min waiting period was allotted to minimize viral spread during needle retractions.

At the completion of the surgical procedures, the dura was closed with silk sutures, the bone opening was covered with Surgicel NU-KNIT (absorbable hemostat), and connective tissues and skin were closed. The animal was removed from anesthesia and placed in a temperature-controlled incubator ventilated with oxygen until full recovery from anesthesia. All animals received Banamine (1 mg/kg for 3 d), dexamethasone (0.5 mg/kg for 3 d), and antibiotic (Rocephin, 25 mg/kg for 7 d) after surgery to prevent pain, edema, and infection, respectively. Behavioral testing began 5 weeks after surgery to allow animals to recover and to provide adequate time for DREADD expression.

### Administration of ligands and plasma assays

All ligands were administered through subcutaneous injection into the interstitial space between the shoulder blades of the animals. CNO was administered at 10 mg/kg body weight, whereas clozapine was administered at 0.1 mg/kg body weight. CNO powder (NIMH C-929, RITI International) was stored at −20°C, protected from light. CNO solutions were freshly prepared on each experimental day, by dissolving the drug in 100% dimethylsulfoxide (DMSO; Sigma-Aldrich) and then diluting with calcium- and magnesium-free PBS (Corning) to a final concentration of 10 mg/ml in 15% DMSO. To prevent degradation due to light exposure, vials and syringes that contained CNO solution were wrapped in aluminum foil. Clozapine powder (Tocris Bioscience) was stored at room temperature, protected from light. Clozapine solutions were also freshly prepared on each day of experiment, using the same dilution methods as described for CNO. Percentages of DMSO and PBS remained constant across all injections to avoid any potential confound that might arise from differences in DMSO concentrations per injection. Vehicle injections consisted of 85% PBS and 15% DMSO.

Animals were trained for awake blood collection from the saphenous vein, following established protocols at the YNPRC (Raper et al., 2014b). On behavioral testing days, blood samples were collected several times after ligand administration to examine plasma concentrations of the ligand during behavioral tasks. For CNO administration, blood was collected at 20, 60, 90, and 135 min postinjection. For clozapine administration, blood was collected at 2, 40, and 60 min postinjection. All blood samples (1–2 ml) were collected in prechilled 2 ml tubes containing EDTA (3.5 mg) and immediately placed on ice. Samples were centrifuged at 2500 rcf for 15 min in a refrigerated centrifuge (at 4°C). Plasma was pipetted off and stored at −80°C until assayed.

All plasma samples were assayed for CNO and clozapine by Precera Bioscience using previously published techniques (Raper et al., 2017). Briefly, liquid chromatography-tandem mass spectroscopy (LC-MS/MS) analyses were performed via reverse-phase chromatography using a Shimadzu Nexera X2 UHPLC system coupled with a QTrap 5500 LC-MS/MS system (Sciex). For plasma samples, 20 μl aliquots of each standard quality control sample, plasma sample, blank, and double blank were transferred to a labeled 96-well plate. Next, 120 μl of 50 ng/ml internal standard (carbamazepine in acetonitrile) was added to standards, quality controls, plasma samples, and blanks, while 120 μl of acetonitrile was added to the double blank, then the plate was centrifuged for 5 min at 3000 rcf. Then, 75 μl of supernatant was transferred into a labeled 96-well plate, and 75 μl of water was added to all samples. Finally, the plate was heat sealed for analysis. The calibration range was 1 − 5000 ng/ml for CNO and 1 − 1000 ng/ml for clozapine.

### Histology and visualization of DREADDs

At the end of the behavioral experiments, the animals received an overdose of pentobarbital and were transcardially perfused with Ringer’s solution, followed by 4% paraformaldehyde and 0.1% glutaraldehyde in phosphate buffer (PB; 0.2 m, pH 7.4). After removing the brain from the skull, we collected vibratome coronal sections (60 µm) in cold PBS (0.01 m, pH 7.4) that were stored at −20° C in an antifreeze solution (30% ethylene glycol/30% glycerol in PB) until further processing.

The staining of Nissl substance was performed to identify the localization of the injection tracks. Following previously published methods (Galvan et al., 2019), the localization of hM4Di was revealed using antibodies against the hemagglutinin (HA) tag, which is fused to hM4Di. We selected brain sections containing or adjacent to the virus solution injection tracks, pretreated with 1% normal goat serum, 1% bovine serum albumin, and 0.3% Triton X-100, and then incubated for 24 h in solutions containing either HA-tag antibodies (1:400; raised in rabbit; clone C29F4; catalog #3724, Cell Signaling Technology; RRID:AB_1549585). As controls, some sections were processed in solutions without the primary antibodies. Incubation in the primary antibodies was followed by 2 h incubation in anti-rabbit secondary biotinylated antibodies (1:200; Vector Laboratories), and then in ABC (avidin–biotin–peroxidase complex) solution (1:200; Vectastain Standard Kit, Vector Laboratories) for 90 min. The sections were then placed in 0.025% 3-3´-diaminobenzidine tetrahydrochloride (Sigma-Aldrich), 0.01 m imidazole (Fisher Scientific) and 0.006% H_2_O_2_ for 10 min. The sections were mounted on slides, coverslipped, and digitized with an Aperio Scanscope CS System (Aperio Technologies).

To estimate the extent of the brain regions expressing the hM4Di, we used a series of HA-tag sections (0.5 mm apart) that encompassed the transfected amygdaloid regions. In digital images (2×), we delineated regions with HA-positive cell bodies, and calculated the area using ImageJ (http://imagej.nih.gov/ij/).

### Behavioral assessments

#### Human intruder paradigm

The human intruder paradigm was chosen because it has been shown to be a robust task for detecting differences in emotional responses of nonhuman primates after adult or neonatal amygdala lesions (Kalin et al., 2004; Machado and Bachevalier, 2008; Raper et al., 2013a,b). The responses of animals to the human intruder were assessed under CNO before transduction, then again under CNO at 1 and 3 months post-transduction, or under vehicle conditions at 2 and 6 months post-transduction. Clozapine activation of the DREADDs was tested at 5 months post-transduction (Fig. 1). On test days when CNO was administered, behavioral testing began ∼80–90 min after injection based on the peak of CNO in CSF in adult rhesus monkeys (Raper et al., 2017). On test days when clozapine was administered, testing began ∼5–10 min after the injection because clozapine readily crosses the blood–brain barrier (Baldessarini et al., 1993). During testing, monkeys were separated from their cagemate, transported to a testing room, and transferred to a stainless steel testing cage (53 × 53 × 55 cm) with one wall made of clear plastic to allow for unobstructed video recording. The human intruder paradigm consisted of three conditions (alone, profile, stare) presented in the same order to all monkeys for a total duration of 30 min. The experimenter wore a rubber mask depicting a different male face at each test, such that both monkeys saw the same novel human intruder at each time point in the study. Other attempts to ensure the novelty of the test throughout the six sessions included the experimenter wearing a different colored laboratory jacket and either different curtains hanging around the room or using a new room to make the environment less familiar. The human intruder test session started with the monkey remaining alone in the cage for 9 min (Alone condition) to acclimatize to the environment and obtain a baseline level of behavior. Then the intruder entered the room and sat 2 m from the test cage, presenting his/her profile to the animal for 9 min (Profile condition or no-eye-contact condition in other experiments). The intruder then left the room while the monkey remained in the cage for a 3 min period, then the intruder re-entered the room, and sat 2 m from the cage, making direct eye contact for 9 min (Stare condition). Emotional behavior responses to the intruder were assessed using Observer XT 14 software (Noldus) and a detailed ethogram (Table 1). One experimenter (J.R.) with a high degree of both intra-rater reliability (Cohen’s κ = 0.98) and inter-rater reliability (Cohen’s κ = 0.86) with other trained experimenters at YNPRC coded all the videotapes. The experimenter was blind to the ligand administration of the animals while coding the videos.

**Table 1.** Behavioral ethogram for human intruder

Category and specific behaviors	Measurement	Brief descriptions
Freeze	Duration	Rigid, motionless posture except slight head movement
Hostile	Cumulative frequency	
Threat bark	Frequency	Low pitch, high intensity, rasping, guttural
Threat	Frequency	Any of the following: open mouth (no teeth exposed), head bobbing, or ear flapping
Cage aggression	Frequency	Vigorously slaps, shakes, or slams body against cage
Lunge	Frequency	A quick, jerky movement toward the stimulus
Vocalizations	Cumulative frequency	
Coo	Frequency	Clear soft pitch and intensity, sounds like “ooooh”
Scream	Frequency	High pitch, high-intensity screech or loud chirp
Anxiety	Cumulative frequency	
Scratch	Frequency	Rapid scratch of body with hands or feet
Body shake	Frequency	Shake of the whole body or just head and shoulders region
Tooth grind^*a*^	Frequency	Repetitive, audible rubbing of upper and lower teeth
Yawn	Frequency	Open mouth widely, exposing teeth
Affiliative^*b*^	Cumulative frequency	
Lipsmack	Frequency	Rapid movement of pursed lips, accompanied by a smacking sound
Present	Frequency	Rigid posture (knees locked) with tail elevated and rump oriented toward the stimulus object
Fearful^*b*^	Cumulative frequency	
Withdrawal	Frequency	Quick, jerky motion away from the stimulus object (jump back)
Grimace	Frequency	Refracted lips, exposed clenched teeth
Stereotypies^*b*^	Cumulative duration	
Pacing	Duration	Repetitive motor pattern around the test cage
Motor stereotypy	Duration	Repetitive, abnormal voluntary or involuntary motor patterns (e.g., swinging, twirling, flipping)

List of all behaviors scored, how they are measured and a brief definition.

*^a^*Behavior for which total duration was also measured.

*^b^*Behavior that was rarely or never seen in the current study.

#### Socioemotional attention task

Damage to the amygdala has been shown to alter attention toward social cues as well as threats in the environment (Aggleton, 2000; Adolphs et al., 2005; Feinstein et al., 2011; Gothard et al., 2018; Payne and Bachevalier, 2019). To measure changes in attention to social and innate aversive stimuli, subjects’ eye movements were monitored using a Tobii T60/T120 Eye Tracker (Tobii Technology). Eye-tracking technology uses noninvasive infrared light reflections on the subjects’ cornea and retina to measure looking behavior. These reflections were calibrated using a 5-point calibration paradigm at the start of each testing session to control for subtle changes in distance and head position. Subjects were acclimated to an infant nonhuman primate chair (Crist Instrument) and positioned 22 inches from the Eye Tracker monitor on which videos were displayed. During test sessions, an experimenter running the Eye Tracker was visually separated from the monkey by a curtain. The experimenter could monitor whether the tracker was detecting the eyes, and a gaze trail was displayed over a view of the presented stimuli on the experimenter’s laptop.

Each test session consisted of a novel set of 54 10 s video clips containing social (36 videos) and nonsocial (18 videos) content. Videos were presented back to back without an intertrial interval between videos clips. All videos were 720 × 480 pixel AVI files, displayed on a 1280 × 1024 resolution monitor (19º × 13º of visual angle) with a refresh rate of 120 Hz. Social videos contained clips of an unfamiliar monkey (4 females, 6 males; age range, 3–20 years) looking toward the camera expressing a threatening, affiliative (lipsmacking), or neutral expression (Mosher et al., 2011, 2014). Videos were controlled for movement of the camera and luminance (Mosher et al., 2011, 2014). Though the size of the face of the monkey in the video varied due to the nature of dynamic video stimuli, the minimum size of the movie monkeys face was 200 × 246 pixels (5º × 6º of visual angle). Nonsocial videos consisted of either aversive stimuli with clips of snakes or spiders, which monkeys innately fear (Mineka et al., 1980; Mineka, 1987; Izquierdo et al., 2005; Chudasama et al., 2009), or neutral stimuli with clips of either enrichment food items (e.g., oranges, grapes, popcorn) or novel items (e.g., train, flower, hot air balloon). Since no head fixation devices were used to restrict the head position to face the monitor, each video was presented twice in each session (to increase the chance that the monkeys would watch the video at least once during the session). Each session lasted between 20 and 30 min. Animals were tested on this socioemotional attention eye-tracking task under CNO at 3.5 months; under vehicle conditions at 3.5, 4, and 5.5 months; or under clozapine at 5 months post-transduction (Fig. 1). Timing between ligand administration and the beginning of behavioral testing was the same as reported for the human intruder task.

Gaze information was recorded at a rate of 60 Hz on a Windows laptop running Tobii Studio software 3.2.2 (Tobii Technology). Several areas of interest (AOIs) were drawn on all video stimuli before data collection using the Tobii Studio software. See Figure 6*A* for illustration of the AOI for Social videos. Regions were defined as eyes (bridge of nose to brow), mouth (nose to chin), face (eyes and mouth), body (outline of monkey body), and background (total viewable movie area). For aversive and neutral videos, regions were defined as foreground (outline of object) and background (total viewable movie area). The primary output measure was fixation duration for each predrawn AOI. For each video, the fixation duration for each AOI was normalized according to the proportion of time spent looking at the corresponding AOI. For example, for the fixation duration in the body AOI was divided by the fixation duration of the total viewable movie area (background), whereas the mouth or eye AOIs were divided by the fixation duration of the entire body AOI. Finally, the average proportion of time spent looking at AOIs was calculated for the first and second presentation for each stimulus type (Social: neutral, lipsmack, threat; or Nonsocial: neutral, aversive).

### Statistical analyses

Pharmacokinetic parameters [i.e., area under the curve (AUC)] for CNO and clozapine were determined using Microsoft Office Excel (Microsoft), and results were graphed using GraphPad Prism 7.02 (GraphPad Software).

For the human intruder paradigm, first we examined whether CNO before transduction impacted behavioral expression on the task by comparing the current data to a group of normally developing controls (*n* = 12; Raper et al., 2013b). To examine the relationship between behavioral responses in the task and the effects of CNO in naive conditions, we used a linear mixed-model analysis, including task conditions (3: Alone, Profile, Stare) and group [2: CNO before transduction in monkeys included in this study, control monkeys from the study by Raper et al. (2013b)] as fixed factors, and individual monkeys as a random factor. To determine whether activation of inhibitory DREADDs could modulate behavioral responses on the human intruder task, we focused our analyses on the condition of the task in which each behavior was most prevalent (see Results). For each behavior (freezing, anxious, hostile, vocalizations), we ran a linear mixed-model analysis with ligand (2: CNO/clozapine, Vehicle) as fixed factor and individual monkeys as a random factor. Data were pooled to compare behavior obtained during typical amygdala activity (before transduction or vehicle sessions) to that obtained during DREADD inhibition of the amygdala (CNO or clozapine). To examine the potential effect of repeated testing on behavioral response, separate linear mixed models were performed for each behavior with test session (1–6) as the fixed factor and individual monkey as the random factor. The first test session was used as a reference category to compare the five subsequent test sessions.

For the socioemotional attention task, we examined whether CNO or clozapine activation of inhibitory DREADDs could modulate looking patterns across social and nonsocial video stimuli. As for the human intruder experiments, data were pooled to compared results under vehicle sessions to those obtained with either CNO or clozapine. For social video stimuli, we used a linear mixed-model analysis with video valence (3: Neutral, Lipsmack, Threat), ligand (2: CNO/clozapine, Vehicle), and presentation Order (2: First or Second presentation) as fixed factors, and individual monkeys as a random factor. This was done for each area of interest separately. For nonsocial video stimuli, we used a linear mixed-model analysis with video valence (2: Neutral, Aversive), ligand (2), and presentation order as fixed factors, and individual monkeys as a random factor. To examine the potential effect of repeated testing on looking patterns, separate linear mixed models were performed for each area of interest with test session (1–5) and valence (2) as the fixed factors and individual monkey as the random factor. The first test session was used as a reference category for all subsequent test session comparisons. Interactions between test session and valence were investigated for each valence type using Welch’s *t* test with test session 1 compared with each subsequent session. All behavioral data were analyzed using SPSS 26 for Windows (IBM), significance was set at *p* < 0.05, and effect sizes were calculated using η_p_^2^.

## Results

### Plasma concentrations of ligands

**Figure 2.**
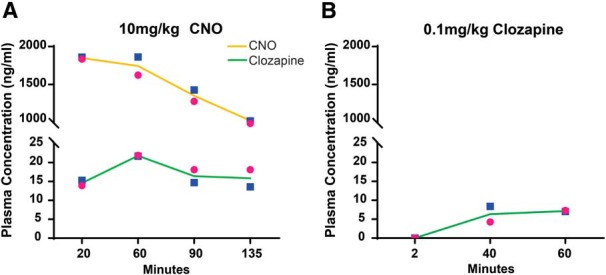
Time–concentration profiles of CNO and clozapine in infant monkeys. ***A***, ***B***, Plasma concentrations of CNO (yellow line) and its metabolite clozapine (green line) following subcutaneous administration of CNO at 10 mg/kg (***A***) and clozapine at 0.1 mg/kg (***B***) in the infant male (blue square) and female (pink circle) monkeys.

Although CNO is the most widely used ligand for activating the DREADDs, it has been shown to have poor penetration of the blood–brain barrier and can be metabolized into clozapine (Gomez et al., 2017; Raper et al., 2017; Allen et al., 2019). Importantly, clozapine has a high affinity for the DREADDs and has been shown to preferentially activate these receptors after low-dose administration (Armbruster et al., 2007; Gomez et al., 2017). To help interpret DREADD-mediated behavioral effects, we collected plasma samples before and after behavioral testing sessions with CNO or low-dose clozapine administrations. The two infant monkeys had similar pharmacokinetic profiles for 10 mg/kg doses of CNO (Fig. 2*A*). Infant plasma concentrations of CNO were high-throughput sample collections with an average AUC value of 171.66 ng/ml/h. As observed previously in adults (Raper et al., 2017), infants also readily metabolized CNO into clozapine, which was detectable starting at 20 min postinjection with an average AUC value of 2.03 ng/ml/h. Since developmental changes in physiology can impact drug pharmacokinetics and pharmacodynamics (Milsap and Jusko, 1994; Lu and Rosenbaum, 2014), it is important to note that the conversion ratio of CNO to clozapine in infant monkeys (1.1%) was similar to that of adult monkeys (1.3%; Raper et al., 2017). Low-dose clozapine (0.1 mg/kg) administration also exhibited a similar pharmacokinetic profile between the two infant monkeys; however, clozapine was not detectable until the second sample collection at 40 min postinjection, and plasma concentrations of clozapine were below levels of converted clozapine after 10 mg/kg CNO administration (Fig. 2*B*). The time course of clozapine levels in plasma was in agreement with previous reports (Baldessarini et al., 1993).

### Structural MRI scans and postmortem verification of DREADD expression

**Figure 3.**
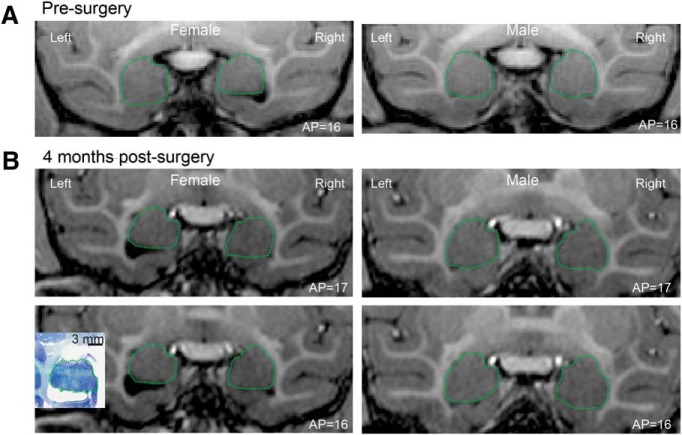
*In vivo* neuroimaging in infant rhesus monkeys. ***A***, ***B***, T1-weighted MRI scan presurgery (***A***) and again at 4 months postsurgery (***B***) for the female and the male monkeys. In the left hemisphere of the female, a reduction in the amygdala was observed at 4 months postsurgery (***B***), which was later confirmed in the Nissl section (inset). The green dashed lines approximately outline the amygdala.

While the presurgical T1-weighted MRI scan did not reveal any structural abnormalities in the monkeys (Fig. 3*A*), a second scan performed at 4 months postsurgery showed a reduction in the volume of the left amygdala in the infant female subject (Fig. 3*B*). Since the right amygdala in the female monkey and both hemispheres in the male monkey looked normal in the postsurgical MRI scan, we presume that the reduced volume was due to a unilateral lesion of the amygdala, likely caused by a hemorrhage or other ischemic event associated with the intracerebral injection surgery, rather than as a result of the DREADD expression. The unilateral lesion was confirmed during histology (Fig. 3*B*, inset). Given the extent of the tissue damage in the amygdala region of the left hemisphere, we were unable to identify the injection sites or the DREADD expression in the left hemisphere of the female subject.

**Figure 4.**
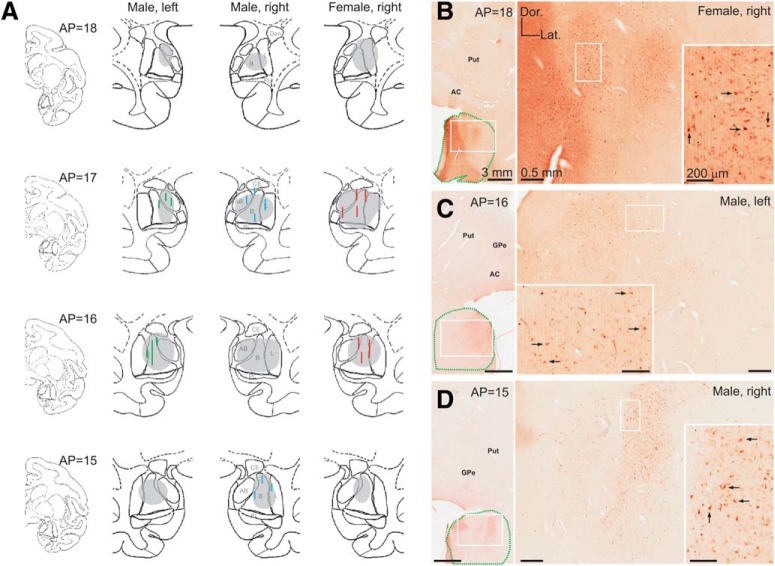
Expression of DREADDs after virus injections in the amygdala. ***A***, Injection tracks (blue lines, male; red lines, female) identified in Nissl stains; and DREADD expression coverage (gray outline) in the amygdala, as identified with immunoperoxidase. ***B–D***, Examples of HA immunoperoxidase labeling in the amygdala. White rectangles indicate areas shown at higher magnification in subsequent panels. Arrows point to examples of HA(hM4Di)-positive neuronal cell bodies. In ***B–D***, the approximate outline of the amygdala is indicated by green dashed lines. Scale bars and orientation in ***B*** apply to ***C*** and ***D***. AP indicates the approximate anteroposterior plane from the interaural line, according to coronal drawings from a normalized infant rhesus monkey brain (J. Bachevalier, unpublished observations). AB, Anterior basal amygdala; AC, anterior commissure; B, basal amygdala; CE, central amygdala; Dor., dorsal; GPe, external segment of the globus pallidus: L, lateral amygdala; Lat., lateral; PL, paralaminar amygdala, Put, putamen.

In Nissl-stained sections, we verified that the virus injection tracks were localized in the amygdala (Fig. 4*A*). To identify the areas with robust DREADD expression, we used antibodies against the HA tag and revealed these antibodies using biotinylated antibodies and immunoperoxidase. In the three hemispheres examined, the HA labeling was found in the basal, lateral, and anterior basal compartments of the amygdala (Fig. 4*A*, gray ovals). The HA-positive area extended ∼4 × 3 × 3 mm in the anteroposterior, dorsoventral, and mediolateral planes, respectively. Figure 4*B–D* shows representative images of brain sections close to the viral vector injection sites in the three hemispheres examined. The HA labeling was observed in numerous cell bodies and proximal dendrites of neurons.

### Modulation of emotional reactivity

**Figure 5.**
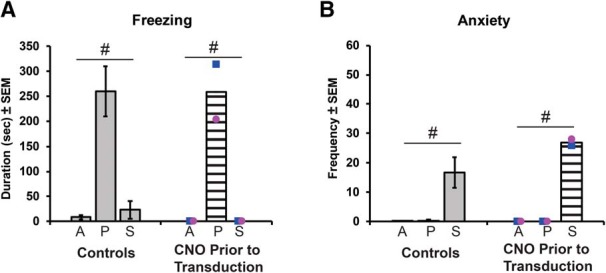
Behavioral responses on the human intruder paradigm before DREADD transduction. ***A***, ***B***, Freezing (***A***) and anxiety (***B***) behaviors measured across task conditions (A, alone; P, profile; S, stare) in the infant female (pink circle) and male (blue square) monkeys before transduction (striped bar), compared with control monkeys from a previous study (historic controls, gray bars; Raper et al., 2013b). #Indicates a significant effect of condition on the task (*p* < 0.05).

To investigate whether CNO administration had effects on the behavior of the animals in the human intruder task before DREADD transduction, their behavior after CNO administration was compared with that exhibited by monkeys of a similar age in a previous study (Raper et al., 2013b). Both groups exhibited increased freezing in the profile condition (Condition: *F*_(2,24)_ = 15.31, *p* < 0.001, η_p_^2^ = 0.56; Fig. 5*A*), increased hostility (Condition: *F*_(2,24)_ = 22.00, *p* < 0.001, η_p_^2^ = 0.65), and increased anxiety in the stare condition (Condition: *F*_(2,24)_ = 10.63, *p* < 0.001, η_p_^2^ = 0.47; Fig. 5*B*), with no interactions or main Group effects (Freezing: *F*_(1,12)_ = 0.05, *p* = 0.83, η_p_^2^ = 0.004; Hostile: *F*_(1,12)_ = 0.13, *p* = 0.73, η_p_^2^ = 0.01; Anxiety: *F*_(1,12)_ = 0.55, *p* = 0.47, η_p_^2^ = 0.04).

As shown in Figure 5 and Table 2, each behavior was most prevalent during one of the human intruder task conditions (i.e., freezing during the Profile condition; and hostile, anxious, and vocalizations during the Stare condition). Thus, the rest of the analyses focused on the condition in which the behavior was more prominent.

**Table 2.** Individual human intruder paradigm data

Session			Freezing (duration)			Anxiety (frequency)			Hostility (frequency)			Vocalizations (frequency)		
order	Ligand	Subject	Alone	Profile	Stare	Alone	Profile	Stare	Alone	Profile	Stare	Alone	Profile	Stare
1	CNO (prior to transduction)	F M	0 0	203.97 313.34	0 0	0 0	0 0	26 28	0 0	0 0	193 151	0 0	0 0	80 41
2	CNO	F M	0 1.63	33.83 170.26	0 0	0 0	0 0	2 15	2 0	0 0	157 158	0 0	0 0	47 45
3	Vehicle	F M	0 0	222.58 342.97	0 0	0 0	0 0	38 71	8 0	0 1	148 112	0 0	0 0	33 19
4	CNO	F M	0 0	40.81 72.9	0 0	0 0	0 0	7 33	4 0	1 0	86 87	0 0	0 0	0 16
5	Clozapine	F M	0 0	53 68.07	0 0	0 0	0 0	0 26	1 0	3 0	147 124	0 3	0 0	116 67
6	Vehicle	F M	0 0	68 250.54	0 0	0 0	0 0	15 32	2 8	1 1	167 62	39 8	162 0	100 48

List of behaviors scored on the human intruder paradigm and the individual values for the female (F) and male (M) infant monkeys across the 6 test sessions and ligand administered during each session.

**Figure 6.**
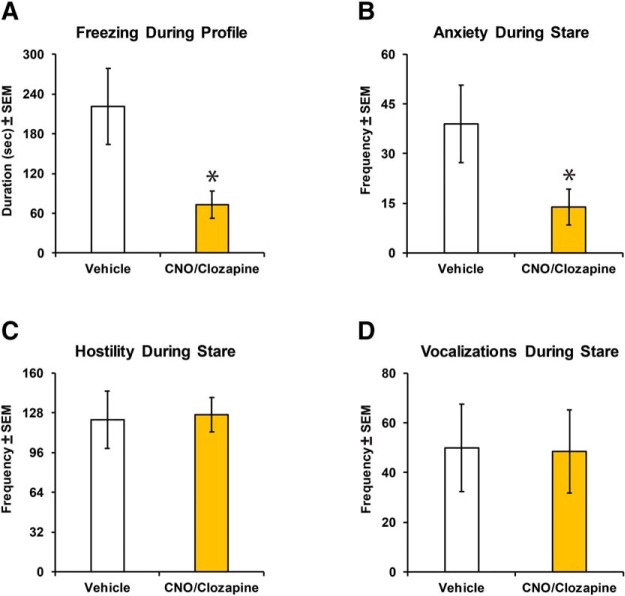
Behavioral responses on the human intruder paradigm with and without DREADD inhibition of the amygdala. ***A–D***, Freezing during the Profile condition (***A***), anxiety (***B***), hostility (***C***), and vocalizations (***D***) during the Stare condition after CNO or clozapine (CNO/clozapine, yellow bars) or vehicle (Veh, open bars) administration. *Indicates a significant effect of ligand (*p* < 0.05).

Once DREADD transduction was completed, inhibition of the amygdala with either CNO or clozapine administration led to decreased freezing during the profile condition of the human intruder task compared with vehicle administration (Ligand: *F*_(1,9)_ = 23.24, *p* = 0.001, η_p_^2^ = 0.72; Fig. 6*A*). DREADD-mediated amygdala inhibition also decreased anxiety expression during the stare condition (Ligand: *F*_(1,9)_ = 7.57, *p* = 0.022, η_p_^2^ = 0.46; Fig. 6*B*). In contrast, DREADD inhibition of the amygdala did not impact hostile behavior expression (Ligand: *F*_(1,9)_ = 0.32, *p* = 0.58, η_p_^2^ = 0.03; Fig. 6*C*) or vocalizations (Ligand: *F*_(1,9)_ = 0.06, *p* = 0.81, η_p_^2^ = 0.01; Fig. 6*D*) during the stare condition. We also examined the effect of repeated testing on behavioral responses. While hostile behavior expression did not differ between the first (CNO before transduction) and the five subsequent test sessions (Test Session: *F*_(1,5)_ = 2.42, *p* = 0.18, η_p_^2^ = 0.33; see Table 2), freezing, anxiety, and vocalizations differed by test session (Test Session: freezing, *F*_(1,5)_ = 9.47, *p* = 0.014, η_p_^2^ = 0.65; anxiety, *F*_(1,5)_ = 8.47, *p* = 0.018, η_p_^2^ = 0.63; vocalizations, *F*_(1,5)_ = 4.99, *p* = 0.05, η_p_^2^ = 0.49). A *post hoc* test revealed decreased freezing between the first session and the sessions when CNO/clozapine was administered (second test session, *p* = 0.018; fourth test session, *p* = 0.007; fifth test session, *p* = 0.007), but not when vehicle was administered (third test session, *p* = 0.62; sixth test session, *p* = 0.079). A *post hoc* test for anxiety revealed a significant increase between the first and third (*p* = 0.018) test sessions, but no difference between the first test session and any other test session. Last, *post hoc* test for vocalizations revealed a significant decrease between the first and fourth (*p* = 0.043) test sessions, but no difference between the first and any other test session.

### Modulation of socioemotional attention

**Figure 7.**
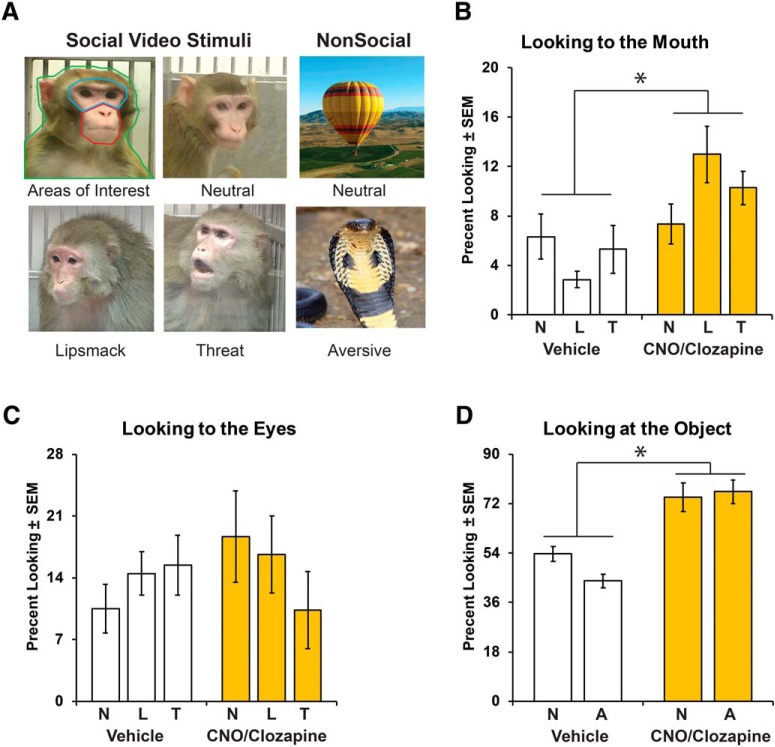
Looking behavior during the socioemotional attention task during DREADD-mediated inhibition of the amygdala. ***A*** represents examples of the video stimuli type, as well as outlines the specific areas of interest (AOIs) mouth (red), eyes (blue), and body (green) for social stimuli. ***B*** and ***C*** illustrate the percentage looking to the mouth or eyes of neutral (N), lipsmack (L), or threatening (T) social videos. ***D*** illustrates the percentage looking at the neutral (N) or aversive (A) nonsocial object after CNO or clozapine (yellow bars) or vehicle (open bars) administration. *Indicates a significant difference from vehicle (*p* < 0.05).

The two infants in this study exhibited gaze fixation durations similar to those found in other studies of non-head-restrained infant monkeys (Parr et al., 2016; Ryan et al., 2019). Compared with vehicle sessions, DREADD activation with either CNO or clozapine administration led to increased attention toward the mouth of conspecifics (Fig. 7*A*; Ligand: *F*_(1,47)_ = 11.17, *p* = 0.002, η_p_^2^ = 0.19) regardless of the emotional valence of the social video stimuli (Ligand × Valence: *F*_(2,47)_ = 2.68, *p* = 0.079, η_p_^2^ = 0.10; Fig. 7*B*). Attention to the mouth did not change with the order of presentation of the videos (Presentation: *F*_(1,47)_ = 1.76, *p* = 0.19, η_p_^2^ = 0.04), and there was no interaction between DREADD-mediated inhibition of the amygdala and presentation order (Ligand × Presentation: *F*_(1,47)_ = 0.92, *p* = 0.34, η_p_^2^ = 0.02). DREADD-mediated inhibition of the amygdala did not impact the amount of time animals spent looking toward the eyes (Fig. 7*C*) or body (not shown) of conspecifics regardless of the emotional valence (Ligand: eyes, *F*_(1,47)_ = 0.43, *p* = 0.52, η_p_^2^ = 0.01; body, *F*_(1,47)_ = 0.80, *p* = 0.38, η_p_^2^ = 0.02; Valence: eyes, *F*_(2,47)_ = 0.35, *p* = 0.71, η_p_^2^ = 0.01; body, *F*_(2,47)_ = 0.47, *p* = 0.63, η_p_^2^ = 0.02). Attention to the eyes and body did change across presentation order (Presentation: *F*_(1,47)_ = 4.68, *p* = 0.036, η_p_^2^ = 0.09; Presentation: *F*_(1,47)_ = 5.48, *p* = 0.024, η_p_^2^ = 0.10, for eyes and body, respectively), such that during the second presentation of stimuli the animals looked at the eyes less and the body more (Table 3). However, DREADD-mediated inhibition of the amygdala did not impact attention to the eyes or body across presentation order (Ligand × Presentation: eyes, *F*_(1,47)_ = 0.33, *p* = 0.57, η_p_^2^ = 0.007; body, *F*_(1,47)_ = 0.004, *p* = 0.95, η_p_^2^ < 0.001). While attention to the eyes did not differ between the first test session (vehicle) and the four subsequent test sessions (Test Session: *F*_(4,44)_ = 1.60, *p* = 0.19, η_p_^2^ = 0.13; Table 3), attention toward the mouth and body significantly differed by test session (Test Session; mouth, *F*_(4,44)_ = 6.26, *p* < 0.001, η_p_^2^ = 0.36; body, *F*_(4,44)_ = 6.22, *p* < 0.001, η_p_^2^ = 0.36). Specifically, *post hoc* test revealed increased attention to the mouth and body between the first and the second test sessions (*p* = 0.003, *p* = 0.008, respectively), the fourth test session (*p* < 0.001, *p* = 0.036, respectively), and the fifth test session (*p* = 0.004, *p* < 0.001, respectively). Attention to any AOI did not differ by stimulus type with repeated testing (Test Session × Valence: eyes, *F*_(8,44)_ = 1.70, *p* = 0.12, η_p_^2^ = 0.24; mouth, *F*_(8,44)_ = 2.03, *p* = 0.06, η_p_^2^ = 0.27; body, *F*_(8,44)_ = 0.71, *p* = 0.68, η_p_^2^ = 0.11).

**Table 3.** Individual data social stimuli

Session			Presentation	Mouth			Eyes			Body		
order	Ligand	Subject	order	Neutral	Lipsmack	Threat	Neutral	Lipsmack	Threat	Neutral	Lipsmack	Threat
1	Vehicle	F	1 2	0.00 0.96	0.00 4.37	0.00 1.84	0.31 19.87	33.33 13.85	37.88 19.04	29.60 45.25	20.63 24.84	0.87 47.21
		M	1 2	0.00 4.99	0.00 7.79	0.00 1.62	3.16 9.11	18.90 13.71	28.67 21.83	24.66 24.55	44.35 19.97	25.72 35.01
2	CNO	F	1 2	7.67 4.35	10.02 10.80	12.90 11.35	32.75 42.77	30.48 22.86	19.42 12.21	48.01 44.36	50.92 57.28	46.71 49.69
		M	1 2	0.29 10.94	4.53 9.26	9.94 13.24	1.75 16.86	8.05 8.97	8.27 6.94	28.50 45.63	28.42 35.98	19.80 41.78
3	Vehicle	F	1 2	2.13 7.86	2.42 4.60	2.87 5.88	9.56 6.76	0.14 10.66	21.73 12.19	33.28 43.95	25.11 44.00	17.54 27.12
		M	1 2	2.24 8.89	1.21 2.28	0.32 9.63	31.28 0.00	20.98 3.33	22.00 0.00	28.70 44.17	35.91 31.07	29.45 28.85
4	Clozapine	F	1 2	15.21 5.57	18.04 35.65	2.18 7.64	30.23 9.14	38.21 6.32	7.74 9.95	45.38 68.35	36.60 36.52	39.91 50.47
		M	1 2	4.76 10.00	3.78 11.87	11.10 13.98	8.37 7.57	12.71 6.24	10.36 8.11	35.92 33.35	27.37 39.65	24.75 25.07
5	Vehicle	F	1 2	19.31 6.82	5.08 3.78	24.09 9.74	9.24 15.06	15.72 16.62	4.17 3.74	60.13 61.65	63.73 61.95	59.86 65.18
		M	1 2	17.23 5.47	0.18 2.60	4.52 3.13	20.40 1.29	17.36 9.36	13.18 1.18	18.91 37.53	23.70 49.22	46.12 48.11

Average percentage looking at specific AOIs for the first and second presentation of social stimuli containing neutral, lipsmack, or threatening emotional valences. Individual values for the female (F) and male (M) infant monkeys across the five test sessions and ligand administered during each session of the Socioemotional Attention task.

For nonsocial videos, DREADD-mediated amygdala inhibition increased attention toward objects (Ligand: *F*_(1,32)_ = 33.62, *p* < 0.001, η_p_^2^ = 0.51; Fig. 7*D*) regardless of the stimulus type (Valence: *F*_(1,32)_ = 0.73, *p* = 0.40, η_p_^2^ = 0.02) or viewing order (Presentation: *F*_(1,32)_ = 1.44, *p* = 0.24, η_p_^2^ = 0.04; Table 4). Repeated testing had an effect on viewing patterns, which depended on the stimulus type (Test Session × Valence: *F*_(4,30)_ = 2.71, *p* = 0.049, η_p_^2^ = 0.27), such that animals viewed both the neutral and aversive stimuli more during the second test session compared with the first test session (Neutral: *t*_(5.25)_ = −2.14, *p* = 0.04; Aversive: *t*_(4.67)_ = −2.97, *p* = 0.02), while no other test sessions differed.

**Table 4.** Individual data nonsocial stimuli

				Object	
Session order	Ligand	Subject	Presentation order	Neutral	Aversive
1	Vehicle	F	1 2	47.60 70.63	38.95 70.22
		M	1 2	28.21 58.27	40.17 67.16
2	CNO	F	1 2	74.61 87.86	87.72 83.51
		M	1 2	58.56 76.47	90.26 69.40
3	Vehicle	F	1 2	54.10 41.48	25.12 27.80
		M	1 2	58.17 54.98	55.99 36.10
4	Clozapine	F	1 2	65.32 66.00	75.28 64.79
		M	1 2	34.11 50.30	66.02 73.88
5	Vehicle	F	1 2	49.33 61.40	26.99 37.21
		M	1 2	68.30 51.33	54.82 46.36

Average percentage looking for the first and second presentation of neutral and aversive nonsocial stimuli. Individual values for the female (F) and male (M) infant monkeys across the 5 test sessions and ligand administered during each session of the Socioemotional Attention task..

## Discussion

This study provides proof of principle that chemogenetic tools can be used in infant nonhuman primates to modulate neuronal activity, resulting in transient and reversible changes in behavior. To date, nonhuman primate chemogenetic studies have focused on adults (Eldridge et al., 2016; Grayson et al., 2016; Nagai et al., 2016; Upright et al., 2018). Considering its reversibility and low invasiveness, this technique holds great promise for developmental studies in which more invasive techniques cannot be used. Our study incorporated the use of two amygdala-dependent behavioral tasks and investigations of the ability of both CNO and low-dose clozapine to activate the DREADD receptors. We have demonstrated that amygdala neurons in infant monkeys can be transduced to express DREADDs after virus solution injections, that the expression is stable for at least 7 months, and that the activation of DREADDs in the neurons modulates some anxiety-related behaviors. We have also shown that the reductive metabolism of CNO to clozapine in infant monkeys is similar to that in adult monkeys and that low doses of clozapine may be sufficient to elicit the DREADD-mediated behavioral effects. Despite its technical limitations (see below), our study provides initial data demonstrating that chemogenetics can be used to investigate developmental behavioral neuroscience research questions.

For this study, we chose the following two tasks that exhibit robust effects after amygdala damage: the human intruder paradigm (Kalin et al., 2004; Machado and Bachevalier, 2008; Raper et al., 2013a,b); and a socioemotional attention task (Adolphs et al., 1994, 2005; Chudasama et al., 2009; Machado et al., 2009, 2011; Hadj-Bouziane et al., 2012; Gothard et al., 2018). Given that CNO can be reductively metabolized into the psychoactive compound clozapine (Gomez et al., 2017; Raper et al., 2017; Allen et al., 2019), it was important to establish that CNO administration alone would not impact behavior before introducing DREADDs into the amygdala. We confirmed that before DREADD transduction in the amygdala, the responses on the human intruder task after CNO administration were similar to those shown in normally developing controls (Fig. 5*A*,*B*; Raper et al., 2013b). Activation of hM4Di DREADDs in the amygdala results in behavioral alterations similar to those seen after both permanent lesion or transient inactivation (Kalin et al., 2001, 2004; Machado and Bachevalier, 2008; Chudasama et al., 2009; Machado et al., 2009; Bliss-Moreau et al., 2011b; Raper et al., 2013a,b; Dal Monte et al., 2015; Wellman et al., 2016). After the viral DREADD transduction, CNO or low-dose clozapine administration reliably decreased freezing and anxiety on the human intruder task. Considering the differences in plasma clozapine levels after CNO or low-dose clozapine administration, the current behavioral effects support the finding that very low doses of clozapine are highly effective for DREADD activation (Gomez et al., 2017). Decreased freezing and anxiety in the human intruder task have been reported following amygdala lesions in nonhuman primates during both adulthood (Kalin et al., 2004; Machado and Bachevalier, 2008) and infancy (Raper et al., 2013a,b). In contrast to results obtained after neonatal amygdala lesions on the human intruder task (Raper et al., 2013a,b), the present study in infant monkeys did not exhibit changes in vocalizations and hostile behavior expression after DREADD-mediated amygdala inactivation. The differences between the previous lesion studies and the current study suggest that lesion-induced plasticity changes after early permanent amygdala damage (Machado et al., 2008; Raper et al., 2014b; Grayson et al., 2017; Payne et al., 2017) result in more extensive behavioral changes compared with the transient chemogenetic inhibition of the amygdala. We cannot completely rule out the possibility that the differences between our results and those of previous lesion studies are due to incomplete silencing of the amygdala using the chemogenetic technique. However, previous lesion studies have shown that even incomplete lesions of the amygdala result in behavioral changes (Kalin et al., 2004; Machado and Bachevalier, 2008; Machado et al., 2009; Raper et al., 2013a,b). In addition, two NHP chemogenetic studies have previously demonstrated that only a small percentage (3–10%) of the target tissue needs to be transduced to produce a behavioral effect (Eldridge et al., 2016; Upright et al., 2018). Thus, it is possible that behavioral effects are evoked even with a partial silencing of the structure.

Amygdala damage has been shown to alter social behavior and attention to social cues (Adolphs et al., 1994; Bauman et al., 2004; Spezio et al., 2007; Raper et al., 2014a; Dal Monte et al., 2015; Wellman et al., 2016; Bliss-Moreau et al., 2017). Lesions of the amygdala have also been shown to decrease fear reactivity and increase visual interest toward aversive stimuli, such as snakes (Kalin et al., 2004; Chudasama et al., 2009; Machado et al., 2009; Bliss-Moreau et al., 2010, 2011a,b; Feinstein et al., 2011). Using an eye-tracking task, we examined how DREADD inhibition of the amygdala altered attention toward social and aversive stimuli. We found that amygdala inhibition with either CNO or clozapine administration resulted in increased looking toward the mouth of novel conspecifics in Social stimulus videos. Our findings of increased looking toward the mouth of Social videos is similar to results obtained after amygdala damage in adult humans and nonhuman primates (Adolphs et al., 1994; Spezio et al., 2007; Hadj-Bouziane et al., 2012; Dal Monte et al., 2015). A recent study found that adult monkeys with neonatal amygdala lesions looked equally to the mouth and eyes of a conspecific, whereas controls attended more to the eyes (Payne and Bachevalier, 2019). Those results are similar to the current study, demonstrating that amygdala inhibition only increased the percentage looking toward the mouth and did not alter looking at the eyes or body of conspecifics. In addition, DREADD-mediated amygdala inhibition resulted in increased looking at Nonsocial videos, including aversive videos containing snakes and spiders. Previous amygdala lesion studies have shown increased interest in aversive stimuli, including increased visual investigation, approach, and manipulation objects (Kalin et al., 2004; Izquierdo et al., 2005; Chudasama et al., 2009; Machado et al., 2009; Bliss-Moreau et al., 2010, 2011a,b; Feinstein et al., 2011). The similarity between the current results and previous lesion research suggests successful chemogenetic inhibition of the amygdala in infant monkeys. In contrast to the differences between DREADD-mediated amygdala inhibition and permanent lesions in response to the human intruder, results from the socioemotional attention task are quite similar to that of permanent lesions (Chudasama et al., 2009; Hadj-Bouziane et al., 2012; Dal Monte et al., 2015). This difference suggests that socioemotional attention is more easily modulated by transient inactivation of the amygdala, whereas behavioral responses, such as hostility, are less sensitive to the temporary DREADD inhibition, as observed during the human intruder task.

There are limitations to this study. First, the study included just two infant monkeys. However, this number of animals is comparable to previous studies using manipulations of neuronal circuits in nonhuman primates (O’Reilly et al., 2013; Eldridge et al., 2016; Papageorgiou et al., 2017; Folloni et al., 2019; Fouragnan et al., 2019). This limitation was also mitigated by the within-subjects design of the study in which the same behaviors were measured several times under vehicle or CNO/clozapine administration. Although repeated testing also has caveats, including the potential to induce behavioral habituation, few statistical differences were detected for test session. Second, we only conducted a presurgery test of CNO on the human intruder task and not the socioemotional attention eye-tracking task. Although, it is possible that CNO/clozapine could have impacted social attention, this is unlikely considering a recent study demonstrating that low-dose clozapine does not impact social behavior or working memory in naive rats (Ilg et al., 2018). Future studies should include testing the DREADD actuators in naive animals during relevant behavioral tasks. Furthermore, although clozapine at the low doses used here preferentially activated DREADDs, we cannot completely rule out contributions of other neurotransmitter receptors; therefore, future studies should investigate the use of novel inert ligands with high affinity for DREADDs (Bonaventura et al., 2018).Third, there was an unintended amygdala lesion in the infant female subject. Although we cannot rule out the possibility that the lesion was caused by the virus injection or the introduction of the artificial DREADD receptors, this is unlikely given that the damage occurred only in one subject and was localized in one hemisphere. Instead, it was most likely due to a hemorrhage or other ischemic event that occurred as a consequence of the intracerebral injections. This event highlights the importance of conducting a postsurgical MRI. Future studies should consider doing a T2-weighted fluid-attenuated inverse recovery MRI sequence within 5–10 d after surgery, which could help to detect edema from a hemorrhage or ischemic event (Nemanic et al., 2002). The extent to which the unilateral lesion impacted behavioral results is unclear; however, previous studies have shown that unilateral amygdala-lesioned monkeys behave similarly to controls (Kalin et al., 2004; Raper et al., 2013b). Therefore, regardless of whether the bilateral silencing of the amygdala was mediated by DREADDs (infant male subject) or achieved by a combination of unilateral lesion and DREADD activation (infant female subject), the transient and reversible effects on behavior were similar between both subjects (Figs. 5, 6, Tables 2–4). Despite its limitations, the current study is the first to demonstrate that chemogenetic tools can be used in young infant nonhuman primates to address developmental behavioral neuroscience questions.

In summary, transient and reversible inhibition of the amygdala is possible in infant monkeys using chemogenetics, specifically hM4Di DREADDs. This study provides initial data demonstrating that chemogenetics can be used in young animals to investigate developmental neuroscience questions that could not previously be investigated due to the limitations of permanent lesions or invasiveness of pharmacological inactivation studies.

## Synthesis

Reviewing Editor: Mark Baxter, Mount Sinai School of Medicine Decisions are customarily a result of the Reviewing Editor and the peer reviewers coming together and discussing their recommendations until a consensus is reached. When revisions are invited, a fact-based synthesis statement explaining their decision and outlining what is needed to prepare a revision will be listed below. The following reviewer(s) agreed to reveal their identity: NONE. Note: If this manuscript was transferred from JNeurosci and a decision was made to accept the manuscript without peer review, a brief statement to this effect will instead be what is listed below.

This manuscript was accepted after editorial review.
